# Diminuição do Sal na Dieta e Melhora na Pressão Arterial: Baixo Custo, Evidências Robustas e Dificuldades na Implementação

**DOI:** 10.36660/abc.20260262

**Published:** 2026-05-06

**Authors:** Paulo César B. Veiga Jardim

**Affiliations:** 1 Universidade Federal de Goiás Faculdade de Medicina Goiânia GO Brasil Faculdade de Medicina da Universidade Federal de Goiás, Goiânia, GO – Brasil; 2 Hospital do Coração de Goiás Goiânia GO Brasil Hospital do Coração de Goiás, Goiânia, GO – Brasil; 3 Academia Goiana de Medicina Goiânia GO Brasil Academia Goiana de Medicina, Goiânia, GO – Brasil

**Keywords:** Cloreto de Sódio na Dieta, Hipertensão, Pressão Arterial

É muito importante vermos o tema da "diminuição do sal na dieta e seus benefícios na pressão arterial" abordado de maneira técnica, reforçando as evidências dos resultados positivos desta prática, sempre demonstrados, mas quase nunca adotados efetivamente.

Primeiro, coloquemos o foco no excelente trabalho de Kelly et al.^1^ Do ponto de vista da qualidade, trata-se de uma Revisão Sistemática e Metanálise realizada com metodologia extremamente cuidadosa, o que torna este artigo mais uma referência sobre um tema de capital importância. Os cuidados tomados pelos autores garantem confiabilidade aos resultados que demonstram, mais uma vez, que diminuir a ingestão de sódio, efetivamente interfere na pressão arterial, de maneira equivalente ao uso de um fármaco anti-hipertensivo. Os autores, de maneira elegante ao concluírem este achado, ainda são cuidadosos no sentido de referir serem necessárias mais informações a este respeito, com estudos maiores, por prazos mais prolongados e que demonstrem benefícios no controle da pressão e em desfechos cardiovasculares.^1^

Mas façamos um passeio pela história e vamos ver o que o tema nos proporciona.

Do ponto de vista epidemiológico, as evidências se acumularam, inicialmente com constatações da relação entre baixa ingestão de sódio e menores valores de pressão arterial em diversas comunidades ao redor do mundo que remontam a meados do século passado. Muitos óbices foram feitos a estes achados, mas nenhum estudo de qualquer natureza mostrou evidências contrárias. E mais, ao longo dos anos foram adicionadas novas evidências, também observacionais, em grupos de indivíduos que modificaram seus hábitos alimentares por mudança de regiões de moradia, com aumento da ingestão de sal e elevações nos valores da pressão.^2-4^

Nos anos 80, MacGregor, que se destacou com diversos estudos sobre o tema, fez uma análise fundamentada, clara e sucinta, da relação entre o consumo do sal e a hipertensão e observou que o conjunto de informações, já era suficiente para que a diminuição do consumo de sal fosse implementado como política pública, dado o baixo custo e a ausência de efeitos adversos.^5^

No início de 1991 é publicado o estudo INTERSALT, que foi outro marco no conhecimento da relação entre consumo de sal e pressão elevada. Desta feita, além desta relação, houve a evidente indicação de que o hábito alimentar da população era inadequado, com excessiva ingestão de sal e este fato, por certo, implicaria em mudanças para cima nos valores da pressão arterial.^6^

No Brasil, também em 1991, Mancilha e Souza e Silva publicam nos ABC, importante e interessante estudo com índios Yanomami, que também fez parte do estudo INTERSALT, demonstrando que aquela população não apresentava nenhum caso de hipertensão, não havia elevação da pressão arterial com a idade e o consumo de sal era bastante inferior ao de populações de regiões industrializadas.^7^

Em 1992 também no ABC, publicamos um artigo relacionado ao tema, onde em comunidade negra isolada (Kalunga), que não fazia uso do sal de adição na alimentação, não encontramos casos de hipertensão, nem elevação significativa da pressão arterial com o aumento da idade.^8^

E finalmente, praticamente fechando o ciclo epidemiológico, tivemos a produção de estudos bem conduzidos demonstrando que a diminuição da ingestão do sal na alimentação, seja com a restrição propriamente dita, seja com o uso de substitutivos do cloreto de sódio, promoveu significativas diminuições da pressão arterial a valores equivalentes ao uso de medicação anti-hipertensiva.^1,9,10^

A soma das evidências ao longo dos anos fez com que as diretrizes mundiais, inclusive a brasileira, orientassem para a redução da ingestão de sódio para no máximo 2 g/dia, o que corresponde a 5 g de sal de cozinha ou 1 colher de chá/dia, e eventualmente fossem utilizados substitutos do sal enriquecidos com potássio.^11-13^

A Organização Mundial da Saúde, que já havia se manifestado, novamente em 2025 publicou suas orientações baseadas nas informações disponíveis, de que é imperativa uma política mundial de estímulo à diminuição do consumo de sal e ao eventual uso de substitutivos do sódio para este fim.^10^

Esperar resultados de estudos de longo prazo com desfechos duros é ser irresponsável e conivente com o risco tão claro evidenciado pela prática mundial do aumento do consumo de cloreto de sódio ano após ano.

Mudar hábitos para a saúde é fundamental, mas de difícil implementação. A educação em saúde passa por gerações e exige políticas públicas muito centradas e continuadas, para que os resultados sejam alcançados. Esta prática deve ser buscada com tenacidade e ao longo do tempo.

Modificações não menos desafiadoras, porém passíveis de resultados em menor intervalo de tempo, passam por uma atuação política no sentido positivo, com uma legislação impositiva que implique em estímulos ou restrições às indústrias alimentícias, de acordo com a adoção ou não, de uma ação efetiva para diminuição do uso do sal na produção dos alimentos que são consumidos em larga escala por toda a população e, mais ainda, por aqueles de menor poder aquisitivo.

A SBC tem trabalhado neste sentido há algum tempo, porém de maneira intermitente. Às vezes de forma mais intensa, mas às vezes, de forma muito sutil, passando até despercebida pela sociedade.

Nós, profissionais de saúde, particularmente os cardiologistas e também nossa Sociedade, necessitamos tornar este tema um "Assunto de Estado", com uma política institucional vigorosa, que permita criar no país um clima semelhante ao que se conseguiu em relação ao consumo de tabaco e propiciou resultados fantásticos em termos da redução de seu consumo.

As informações científicas estão aí há mais de meio século, chega de espera, chega de atitudes contemplativas.

É hora da ação.
