# Supplementary material for: Diminuição do Sal na Dieta e Melhora na Pressão Arterial: Baixo Custo, Evidências Robustas e Dificuldades na Implementação
Source: Arq Bras Cardiol. 2026 May 6;123(3):e20260252. [Article in Portuguese] doi: 10.36660/abc.20260262 (PMC13398867; doi:10.36660/abc.20260262)
Supplement: Translation

# Translation: Reduction of Salt in the Diet and Improvement in Blood Pressure: Low Cost, Robust Evidence, and Challenges in Implementation

**DOI:** 10.36660/abc.20260262i

**Authors:** Paulo César B. Veiga Jardim

**Affiliations:** 1 Goiânia GO Brazil Faculdade de Medicina da Universidade Federal de Goiás, Goiânia, GO – Brazil; 2 Goiânia GO Brazil Hospital do Coração de Goiás, Goiânia, GO – Brazil; 3 Goiânia GO Brazil Academia Goiana de Medicina, Goiânia, GO – Brazil

**Keywords:** Dietary Sodium Chloride, Hypertension, Arterial Pressure

We must see the topic of "reducing salt in the diet and its benefits on blood pressure" addressed technically, thereby reinforcing the evidence of the positive results of this practice, which are always demonstrated but almost never effectively adopted.

First, let us focus on the excellent work by Kelly et al.^1^ From a quality standpoint, this is a Systematic Review and Meta-Analysis conducted with extremely careful methodology, making this article yet another reference on this crucial topic. The care taken by the authors ensures the reliability of the results, which demonstrate, once again, that reducing sodium intake effectively affects blood pressure in a manner equivalent to the use of an antihypertensive drug. The authors, elegantly concluding this finding, are also careful to point out that more information is needed in this regard, with larger studies, over longer periods, and demonstrating benefits in blood pressure control and cardiovascular outcomes.^1^

But let us take a walk through history and see what the topic offers us.

From an epidemiological point of view, evidence has accumulated, initially with findings of a relationship between low sodium intake and lower blood pressure values in various communities around the world dating back to the mid-20th century. Many objections were raised to these findings, but no study of any kind has shown contrary evidence. Furthermore, over the years, new evidence, also observational, has been added in groups of individuals who modified their eating habits due to changes in their residential areas, with increased salt intake and elevations in blood pressure values.^2-4^

In the 1980s, MacGregor, who distinguished himself with several studies on the subject, conducted a well-founded, clear, and concise analysis of the relationship between salt consumption and hypertension, and observed that the body of information was already sufficient for the reduction of salt consumption to be implemented as a public policy, given the low cost and the absence of adverse effects.^5^

In early 1991, the INTERSALT study was published, which was another milestone in understanding the relationship between salt consumption and high blood pressure. This study, in addition to establishing this relationship, clearly indicated that the population's dietary habits were inadequate, with excessive salt intake, and this fact would certainly lead to upward changes in blood pressure values.^6^

In Brazil, also in 1991, Mancilha and Souza e Silva published in the ABC, an important and interesting study with Yanomami Indians, which was also part of the INTERSALT study, demonstrated that this population did not present any cases of hypertension, there was no increase in blood pressure with age, and salt consumption was significantly lower than that of populations in industrialized regions.^7^

In 1992, also in ABC, we published an article related to the topic, where in an isolated black community (Kalunga), which did not use added salt in its diet, we found no cases of hypertension, nor a significant increase in blood pressure with increasing age.^8^

And finally, practically closing the epidemiological cycle, we had the production of well-conducted studies demonstrating that reducing salt intake in the diet, whether through actual restriction or the use of sodium chloride substitutes, promoted significant decreases in blood pressure to values equivalent to the use of antihypertensive medication.^1,9,10^

The accumulation of evidence over the years has led global guidelines, including those in Brazil, to recommend reducing sodium intake to a maximum of 2g/day, which corresponds to 5g of table salt or 1 teaspoon/day, and possibly using potassium-enriched salt substitutes.^11-13^

The World Health Organization, which had already expressed its views, again published its guidelines in 2025 based on available information, stating that a global policy to encourage the reduction of salt consumption and the eventual use of sodium substitutes for this purpose is imperative.^10^

To expect results from long-term studies with hard outcomes is to be irresponsible and complicit in the clearly evidenced risk, demonstrated by the worldwide practice of increasing sodium chloride consumption year after year.

Changing habits for better health is essential, but difficult to implement. It spans generations and requires highly focused and continuous public policies to achieve results. This practice must be pursued tenaciously and over time.

Modifications that are no less challenging, but capable of yielding results in a shorter timeframe, involve positive political action, with mandatory legislation that implies incentives or restrictions for the food industry, depending on whether or not effective action is taken to reduce salt use in the production of foods that are consumed on a large scale by the entire population, and even more so by those with lower purchasing power.

The SBC has been working in this direction for some time, albeit intermittently. Sometimes more intensely, but sometimes, in a very subtle way, it even goes unnoticed by society.

We, healthcare professionals, particularly cardiologists, and also our Society, need to make this issue a "Matter of State," with a vigorous institutional policy that allows us to create a climate in the country similar to that which was achieved in relation to tobacco consumption and yielded fantastic results in terms of reducing its use.

The scientific information has been available for over half a century; enough waiting, enough contemplative attitudes.

It's time for action.
